# Efficacy and Safety of Pirfenidone for Mitigation of Interstitial Lung Abnormalities in COVID‐19 Patients: A Meta‐Analysis

**DOI:** 10.1155/carj/8812779

**Published:** 2026-01-10

**Authors:** Ziyi Zhang, Xiang Fang, Jinhui Gao, Heming Sun, Ting Xu, Jiajia Wang

**Affiliations:** ^1^ Department of Pulmonary and Critical Care Medicine, The First Affiliated Hospital of Soochow University, Suzhou, 215000, China, sdfyy.cn; ^2^ Suzhou Medical College, Soochow University, Suzhou, 215123, China, scu.edu.tw; ^3^ Department of Public Health, The Fourth Affiliated Hospital of Soochow University, Suzhou, China

**Keywords:** COVID-19, glucocorticoid, interstitial lung abnormalities, pirfenidone

## Abstract

**Background:**

Although post‐COVID‐19 interstitial lung abnormalities (ILAs) are common, the use of antifibrotic agents to prevent their onset and progression is controversial. We aimed to investigate the effectiveness and safety of pirfenidone to mitigate the onset and progression of ILAs in patients with severe COVID‐19.

**Methods:**

We systematically searched literature published before July 21, 2025, from PubMed, Embase, Cochrane Library, Web of Science, China National Knowledge Infrastructure, China Biology Medicine, Weipu, and Wanfang databases, without language limitation. Randomized controlled trials and cohort studies that evaluated the effect of pirfenidone on COVID‐19–induced ILAs were included. Risk of bias was determined using the Revised Cochrane Randomized Trial Risk Bias Tool Version 2 and the Newcastle‐Ottawa Scale. The efficacy and safety of pirfenidone for ILAs in COVID‐19 were analyzed by Review Manager 5.4 software.

**Results:**

Eight studies were included, comprising 335 patients in pirfenidone treatment groups and 302 controls. Risk of bias ranged from low to moderate. Pirfenidone significantly decreased chest high‐resolution CT (HRCT) scores during early‐ and late‐stage COVID‐19 and significantly improved forced expiratory volume in 1 s, especially in late‐stage COVID‐19. Pirfenidone treatment was associated with statistically nonsignificant trends toward improved forced vital capacity and decreased all‐cause mortality. Furthermore, HRCT scores, pulmonary function, and inflammatory cytokine levels following pirfenidone treatment were superior to those obtained after glucocorticoid therapy. The incidence of gastrointestinal adverse events was higher in the pirfenidone than the control group, but no serious adverse events or fatalities occurred.

**Conclusion:**

Pirfenidone therapy may mitigate ILAs and preserve pulmonary function among survivors of COVID‐19 pneumonia. Furthermore, pirfenidone exhibited acceptable safety and tolerability profiles.

**Highlights**

1. We report the first meta‐analysis of pirfenidone therapy to mitigate post‐COVID‐19 ILAs.
2. Pirfenidone may outperform glucocorticoid therapy in reducing inflammation and COVID‐19 sequelae.
3. Pirfenidone therapy should be considered for patients with early‐ and late‐stage COVID‐19 pneumonia.

## 1. Introduction

The COVID‐19 pandemic caused more than 778 million cases and over 7 million deaths between its first report in December 2019 and October 19, 2025, and is still prevalent in the global population (World Health Organization, https://www.who.int/). Long‐term pulmonary complications of COVID‐19 have raised increasing concern. Two prospective observational studies disclosed that 71.3%–96.8% of surviving patients hospitalized with COVID‐19 pneumonia exhibited CT findings suggesting interstitial lung disease (ILD) at 1‐year follow‐up [1, 2]. Persistent radiographic abnormalities are closely associated with impaired lung function, high risk of mortality, and poor quality of life [3, 4]. Although some COVID‐19 patients with post‐COVID‐19 interstitial lung abnormalities (ILAs; the radiographic signature of ILD) show partial or complete resolution, the majority experience a chronic course [5, 6]. Considering the global scale of the COVID‐19 pandemic, the mitigation of COVID‐19–induced ILD represents a significant issue for all populations.

SARS‐CoV‐2 triggers profibrotic macrophage responses and pronounced fibroproliferative ARDS. The pathogenesis of COVID‐19–induced pulmonary fibrosis is characterized by persistent inflammation and excessive myofibroblast proliferation secondary to diffuse alveolar damage that are similar to the mechanisms of idiopathic pulmonary fibrosis (IPF) development [7, 8].

Pirfenidone, a novel antifibrotic agent, exerts anti‐inflammatory, antifibrotic, and antioxidant activities; is an effective treatment of IPF; and has an established safety profile [9]. Multiple studies have demonstrated that pirfenidone may attenuate the decline in lung function by approximately 50% and lower the relative risk (RR) of death by about 48% in IPF patients [10, 11].

Therefore, pirfenidone has been evaluated for the prevention of the development and progression of ILD in COVID‐19 patients. However, the clinical benefit of pirfenidone in treating ILD following severe COVID‐19 remains controversial. In some clinical settings, pirfenidone alleviated COVID‐19–induced ILAs [12, 13]. However, several other studies demonstrated that the post‐COVID‐19 ILAs may resolve spontaneously, and that prolonged, low‐dose glucocorticoid therapy may have been beneficial [14–16]. This study aimed to comprehensively explore the efficacy and safety of pirfenidone in preventing the onset and progression of COVID‐19–induced ILAs. Furthermore, we compared the outcomes of pirfenidone and glucocorticoid therapies.

## 2. Methods

This meta‐analysis was conducted in accordance with the Preferred Reporting Items for Systematic Reviews and Meta‐Analyses (PRISMA) guideline [17] and was registered with PROSPERO (CRD42024569389).

### 2.1. Information Sources and Search Strategy

Two investigators conducted a systematic literature search across PubMed, Embase, Cochrane Library, Web of Science, China National Knowledge Infrastructure, China Biology Medicine, Weipu, and Wanfang databases. The following keywords combinations were applied in the above databases: “COVID‐19” or “2019 novel coronavirus disease” or “2019‐nCoV Infection” or “SARS‐CoV‐2” or “Severe Acute Respiratory Syndrome Coronavirus 2 Infection” or “COVID‐19 Pandemic” AND “pulmonary fibrosis” or “lung fibrosis” or “interstitial pneumonitis” or “interstitial lung disease” or “interstitial pulmonary disease” or “ILDs” or “interstitial pneumonia” AND “antifibrotic agents” or “antifibrotic therapy” or “antifibrotic treatment” or “antifibrotic medicine” or “antifibrotic drug” or “pirfenidone.” The search strategy is presented in detail in Supporting Table S1. There was no language restriction, and the search timeline extended from the inception of these databases to July 21, 2025. The investigators also conducted a manual search to review the citations of selected articles to ensure that all relevant studies were retrieved. Randomized controlled trials (RCTs) and prospective cohort studies comparing the efficacy and safety of pirfenidone versus any comparator in COVID‐19–induced ILAs were considered. After removing duplicates, two investigators independently accomplished the assessment of eligible articles by reviewing the titles, abstracts, and keywords. The final selection of studies depended on full‐text screening. Differences were resolved by consultation until consensus was reached.

### 2.2. Study Selection

Inclusion criteria were as follows. (1) Patients were aged more than 18 years. (2) The diagnosis of COVID‐19 was confirmed by a positive result for SARS‐CoV‐2 RNA detected by a polymerase chain reaction (PCR) test. (3) Patients had no medical history of pre‐COVID‐19 pulmonary fibrosis or interstitial pneumonitis. (4) COVID‐19 patients in the treatment groups received pirfenidone, while those in control groups were treated with other comparators (e.g., placebo, standard therapy, corticosteroids, and N‐acetylcysteine). (5) Outcomes included at least one of the following: imaging findings, pulmonary function parameters, mortality, length of hospital stay (LOHS), or adverse reactions.

The following were excluded. (1) Studies that lacked a control group or adequate data. (2) Literature reviews, case reports, letters, notes, or editorials. (3) Incomplete studies and nonhuman trials. (4) Studies that included pregnant or lactating females.

### 2.3. Data Extraction and Quality Assessment

Extraction of required data was performed independently by two investigators according to the established data extraction table, and discrepancies were resolved by negotiation. Authors were contacted to obtain missing or unclear data. The extracted data included the following. (1) Basic study information: first author, publication year, study design, disease stage, intervention, and outcomes. (2) Demographic features: sample size, mean age, and sex ratio. (3) Outcome indices: forced vital capacity (FVC), forced expiratory volume in the first second (FEV1), percutaneous arterial oxygen saturation (SpO_2_), cytokine level, chest high‐resolution computed tomography (HRCT) scores, LOHS, all‐cause mortality, and incidence of adverse events. Baseline and change values for HRCT scores, FVC, FEV1, SpO_2_, and cytokine levels were included. We assessed the risk of bias in RCTs independently by using the Revised Cochrane Randomized Trial Risk Bias Tool Version 2 (RoB2) in five domains and derived the overall bias. The summary results with high risk, some concerns, or low risk were displayed in red, yellow, or green, respectively. Meanwhile, the quality of the cohort study was assessed by the Newcastle‐Ottawa Scale (NOS). The scale assessed studies primarily in three domains: sample selection, cohort comparability, and outcome evaluation. Articles with 6 stars or more were considered high‐quality articles. In addition, because the number of studies included was less than 10, the funnel plot was deemed insufficient to assess publication bias. Instead, we employed Egger’s test and sensitivity analyses to assess the risk of bias and robustness of results. Sensitivity analyses were accomplished by removing one study at a time and investigating the impact of a single study on the overall risk estimate. Additionally, the risk of publication bias was also assessed qualitatively by examining the comprehensiveness of our search for gray literature and the consistency of the published results.

### 2.4. Definition of Early‐Stage and Late‐Stage COVID‐19

Early‐stage COVID‐19 was defined by a disease duration of less than two weeks after COVID‐19 diagnosis and the absence of definite ILAs on CT scan [21]. Late‐stage COVID‐19 was defined by a disease duration of more than two weeks after COVID‐19 diagnosis or CT findings of ILAs [18].

### 2.5. Statistical Analysis

Statistical analysis was performed by using the up‐to‐date software Review Manager 5.4 (Nordic Cochrane Centre). Continuous variables were analyzed using the inverse variance method to calculate the mean difference (MD) with 95% confidence intervals (CIs). If the metrics of variables differed, we calculated the standard mean difference (SMD) with 95% CIs. Dichotomous variables were implemented by the Mantel–Haenszel method to present RR and 95% CIs as effect measures. The Q‐test was used to qualitatively assess heterogeneity among studies using the *P* value. A *P* value > 0.1 indicated homogeneity, but otherwise suggested heterogeneity. Moreover, heterogeneity was measured quantitatively by the *I* ^2^ test. *I* ^2^ values > 75%, > 50%, and < 25% indicated high, moderate, and low heterogeneity, respectively. When heterogeneity was present, the random effect model was applied to pool the data; conversely, the fixed‐effects model was used. Subgroup analysis was conducted based on the different stages of COVID‐19. The results were shown in a forest plot.

## 3. Results

After using the subject and entry terms obtained from the Medical Subject Headings (MeSH) vocabulary, a total of 1122 articles were identified in the databases. A total of 472 and 650 articles were retrieved from Chinese‐language and English‐language databases, respectively, while 5 studies were searched manually after reviewing the references of included literature. We initially screened the titles and abstracts of 708 selected articles based on inclusion and exclusion criteria and chose 113 for full‐text screening. Ultimately, we included eight studies [19–26] with no disagreement, comprising five RCTs and three cohort studies (Table 1) that included 637 participants (335 in experimental groups and 302 in control groups). A flowchart that illustrates the study selection process is provided in Supporting Figure S1. Among these eight studies, two evaluated patients presenting with early‐stage COVID‐19 [21, 24]; five studies included patients with late‐stage disease [19, 20, 23, 25]; and one did not specify disease stage [22]. The baseline characteristics of participants across all studies were comparable, presenting no statistical difference between the experimental and control groups (*p* > 0.05). Basic characteristics of the included studies are summarized in Table 1. Among the included RCTs, four [21, 22, 25, 26] were assessed at low risk of bias and one [24] at some concern of bias following RoB‐2 evaluation (Figures S2 and S3). All three cohort studies evaluated by the NOS scale were assessed to be of high quality, with seven stars for two studies [20, 23] and six stars for one study [19] (Supporting Table S2). Sensitivity analysis demonstrated that no individual study significantly altered the overall risk estimation. Egger’s test found no significant publication bias (all *p* values > 0.05).

**Table 1:** Basic characteristics of included studies.

Author	Year	Study design	Disease stage	Experimental group (n)	Control group (n)	Outcome
Acat [19]	2021	Cohort study	Late	Pirfenidone + standard treatment (13)	Standard treatment (9)	1, 3, 5,6,7,9
Singh [20]	2022	Cohort study	Late	Pirfenidone (30)	Glucocorticoid (30)	7, 10, 12
Boshra [21]	2022	RCT	Early	Pirfenidone + standard treatment (47)	Standard treatment (53)	7, 9, 10, 11
Zhang [22]	2022	RCT	Unknown	Pirfenidone + standard treatment (73)	Standard treatment (73)	5, 6, 7, 8, 9, 10, 11, 12
Banerjee [23]	2022	Cohort study	Late	Pirfenidone (35)	Placebo (35)	1, 2, 3, 4
Tanvir [24]	2022	RCT	Early	Pirfenidone (28)	Glucocorticoid (28)	1, 2, 5, 6, 7, 10, 12
Chen [25]	2024	RCT	Late	Pirfenidone + standard treatment (40)	NA + standard treatment (40)	1, 2, 3, 4, 7, 8
Peloche [26]	2025	RCT	Late	Pirfenidone (69)	Placebo (34)	4,7,11,12

*Note:* Standard treatment included antiviral, antibiotic, anticoagulant, glucocorticoid, and oxygen therapies. Outcome: 1. Forced expiratory volume in 1 second (FEV1); 2. Absolute change in FEV1 level (ΔFEV1) ; 3. Forced vital capacity (FVC); 4. Absolute changes in FVC (ΔFVC) ; 5. Percutaneous arterial oxygen saturation (SpO2); 6. Absolute changes in SpO2 (ΔSpO2); 7. Change in HRCT score; 8. Absolute change in TNF‐α level (ΔTNF‐α); 9. Length of hospital stay (LOHS); 10. All‐cause mortality; 11. Gastrointestinal adverse events; 12. Discontinuation due to adverse events.

Abbreviations: NA = N‐acetylcysteine; RCT = randomized controlled trial.

### 3.1. Effect of Pirfenidone on Chest HRCT Scores

**Figure 1:**
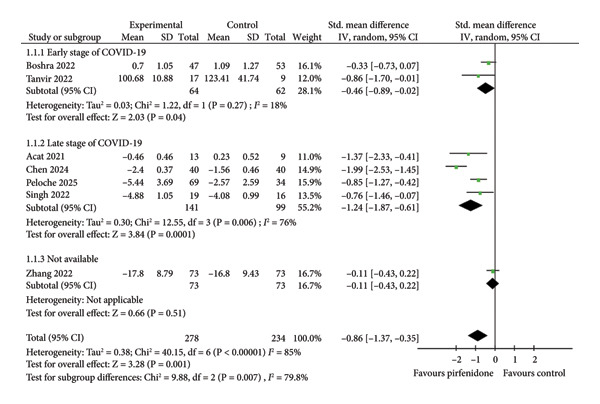
Comparison of HRCT scores between pirfenidone and control groups.

Seven studies [19–22, 24–26] enrolling 278 patients in the pirfenidone group and 234 patients in control group reported mean chest HRCT scores before and after treatment. The data extraction Excel calculation table was used to compute the absolute standard change of HRCT scores from the baseline value. Because of high interstudy heterogeneity (*I* ^2^ = 85%, *p* < 0.001) (Figure 1), the random effect model with standardized effect size was employed to combine the data. Pirfenidone therapy significantly decreased HRCT scores as compared to the control group (SMD = −0.86; 95% CI: −1.37 to −0.35; *p* = 0.001) (Figure 1). Subgroup analysis confirmed the effect in both early‐ and late‐stage COVID‐19 (SMD = −0.46; 95% CI: −0.89 to −0.02; *p* = 0.04, and SMD = −1.24; 95% CI: −1.87 to −0.61; *p* < 0.001) (Figure 1).

### 3.2. Effect of Pirfenidone on Pulmonary Function, SpO_2_, Inflammatory Cytokines, and Prognosis

#### 3.2.1. Effect on FEV1

Four studies [19, 23–25] enrolling 105 patients in the pirfenidone group and 93 patients in the control group compared the changes of FEV1 between the two groups. Because of moderate interstudy heterogeneity (*I* ^2^ = 51%, *p* = 0.10) (Figure 2(a)), the random effect model with a standardized effect size was employed to combine the data. Pirfenidone treatment improved FEV1 when compared to the control group (SMD = 0.94; 95% CI: 0.48 to 1.40; *p* < 0.001) (Figure 2(a)). Subgroup analysis disclosed that pirfenidone therapy significantly improved FEV1 in late‐stage COVID‐19 (SMD = 1.04; 95% CI: 0.50 to 1.58; *p* < 0.001) (Figure 2(a)). However, the effect of pirfenidone on FEV1 during early‐stage disease was difficult to evaluate because it was addressed by only one study (SMD = 0.51; 95% CI: −0.32 to 1.33; *p* = 0.23) (Figure 2(a)).

Figure 2Comparisons of FEV1 and ΔFEV1 between pirfenidone and control groups.

**Figure:**
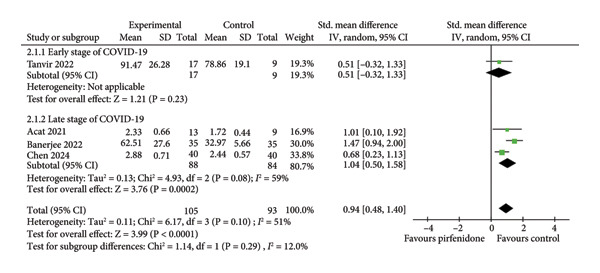
(a)

**Figure:**
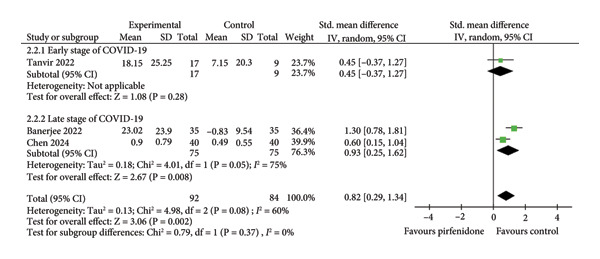
(b)

Three studies [23–25] enrolling 92 patients in the pirfenidone group and 84 patients in the control group reported absolute changes in FEV1 (ΔFEV1) before and after treatment. The data extraction Excel calculation table was used to compute the absolute standard change of FEV1 from the baseline value. Because of moderate interstudy heterogeneity (*I* ^2^ = 60%, *p* = 0.08) (Figure 2(b)), the random effect model with standardized effect size was employed to combine the data. Pirfenidone therapy significantly increased ΔFEV1 (SMD = 0.82; 95% CI: 0.29 to 1.34; *p* = 0.002) (Figure 2(b)). Subgroup analysis indicated that pirfenidone was effective during late‐stage COVID‐19 (SMD = 0.93; 95% CI: 0.25 to 1.62; *p* = 0.008). The effect of pirfenidone on ΔFEV1 change during early‐stage disease was difficult to evaluate due to the sole study listed (SMD = 0.45; 95% CI: −0.37 to 1.27; *p* = 0.28) (Figure 2(b)).

#### 3.2.2. Effect on FVC

Four studies [19, 23, 25, 26] enrolling 157 patients in the pirfenidone group and 118 patients in the control group investigated posttreatment FVC. All three studies addressed late‐stage disease. Because of high interstudy heterogeneity (*I* ^2^ = 78%, *p* = 0.004) (Figure 3(a)), we used a random effect model with a standardized effect size to combine the data. Pirfenidone treatment was associated with a trend toward improved FVC values with statistical significance (SMD = 0.58; 95% CI: 0.02 to 1.14; *p* = 0.04) (Figure 3(a)).

Figure 3Comparisons of FVC and ΔFVC between pirfenidone and control groups.

**Figure:**
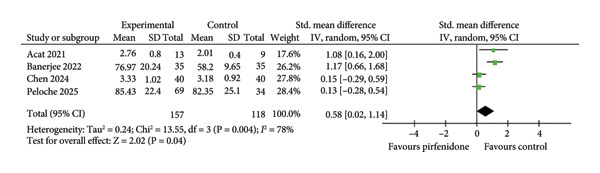
(a)

**Figure:**
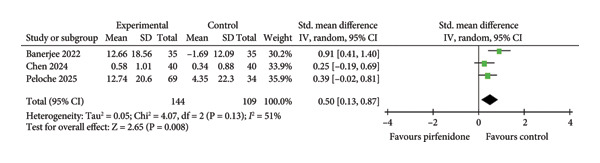
(b)

Three studies [23, 25, 26] regarding late‐stage COVID‐19 enrolled 144 patients in the pirfenidone group and 109 patients in the control group and revealed absolute changes in FVC (ΔFVC) pre‐ and posttreatment. We employed the data extraction Excel calculation table to compute the absolute standard change of FVC from the baseline value. Because of moderate interstudy heterogeneity (*I* ^2^ = 51%, *p* = 0.13) (Figure 3(b)), we used a random effect model with a standardized effect size to combine the data. Pirfenidone treatment was associated with a significantly improved ΔFVC (SMD = 0.50; 95% CI: 0.13 to 0.87; *p* = 0.008) (Figure 3(b)).

#### 3.2.3. Effect on SpO_2_

Three studies [19, 22, 24] enrolling 103 patients in the pirfenidone group and 91 patients in the control group compared changes in SpO_2_ after treatment of early‐stage COVID‐19. Because of low interstudy heterogeneity (*I* ^2^ = 0%, *p* = 0.96) (Supporting Figure S4A), we used a fixed effect model with a standardized effect size to combine the data. Compared with the control group, SpO_2_ was not significantly increased after pirfenidone treatment (SMD = 0.18; 95% CI: −0.11 to 0.46; *p* = 0.22) (Supporting Figure S4A). The same three studies reported absolute changes in pre‐ and posttreatment SpO_2_ levels (ΔSpO_2_). The data extraction Excel calculation table was used to compute the ΔSpO_2_ from the baseline value. Because of low interstudy heterogeneity (*I* ^2^ = 0%, *p* = 0.71) (Supporting Figure S4B), we used a fixed effect model with a standardized effect size to combine the data. ΔSpO_2_ values were similar between the pirfenidone and control groups (SMD = 0.17; 95% CI: −0.11 to 0.46; *p* = 0.23) (Supporting Figure S4B).

#### 3.2.4. Effect of Pirfenidone on Inflammatory Cytokines

**Figure 4:**
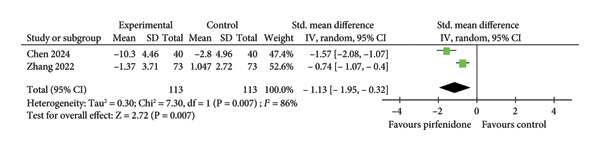
Comparison of ΔTNF‐α level between pirfenidone and control groups.

Two studies [22, 25] enrolling 113 patients in the pirfenidone group and 113 patients in the control group reported the absolute change in pre‐ and posttreatment TNF‐α (ΔTNF‐α) levels. The data extraction Excel calculation table was used to compute ΔTNF‐α. Because of significant interstudy heterogeneity (*I* ^2^ = 86.0%; *p* = 0.007) (Figure 4), the random effect model was used to pool the data. Pirfenidone treatment significantly decreased TNF‐α level in the experimental group (SMD = −1.13; 95% CI: −1.95 to −0.32; *p* = 0.007) (Figure 4).

#### 3.2.5. Effect of Pirfenidone on LOHS

Three studies [19, 21, 22] enrolling 133 patients in the pirfenidone group and 135 patients in the control group investigated LOHS. Because of moderate interstudy heterogeneity (*I* ^2^ = 69%, *p* = 0.04) (Supporting Figure S5), the random effect model was employed to combine the data. Pirfenidone therapy did not decrease the LOHS when compared to the control group (MD = −1.47; 95% CI: −5.07 to 2.13; *p* = 0.42) (Supporting Figure S5).

#### 3.2.6. Effect of Pirfenidone on All‐Cause Mortality

Four studies [20–22, 24] enrolling 178 patients in the pirfenidone group and 184 patients in the control group investigated all‐cause mortality. Because of low interstudy heterogeneity (*I* ^2^ = 16%, *p* = 0.31) (Supporting Figure S6), the fixed effect model was employed to combine the data. Pirfenidone therapy resulted in a trend toward reduced all‐cause mortality that did not reach statistical significance as compared to the control group (RR = 0.62; 95% CI: 0.37 to 1.03; *p* = 0.07) (Supporting Figure S6).

### 3.3. Comparison of Pirfenidone and Corticosteroid Therapies

#### 3.3.1. Effect on Chest HRCT Scores and TNF‐α Level

Patients who received glucocorticoid were enrolled in the control group, and patients who received either pirfenidone or pirfenidone combined with glucocorticoid were enrolled in the experimental group (Table 1). Notably, experimental groups exhibited significantly decreased chest HRCT scores and TNF‐α level compared to control groups (Figures 1 and 4), indicating that pirfenidone therapy outperformed corticosteroid treatment in the reduction of inflammation and COVID‐19–induced ILAs.

#### 3.3.2. Effect on FEV1

Three studies [19, 24, 25] compared changes of FEV1 between experimental groups (total of 70 patients) that received either pirfenidone or pirfenidone combined with glucocorticoid (Table 1) and control groups (total of 58 patients) that received glucocorticoid treatment. The fixed effect model with a standardized effect size was employed to combine the data due to low interstudy heterogeneity (*I* ^2^ = 0%, *p* = 0.61) (Figure 5(a)). Compared to the control groups, the experimental groups exhibited markedly improved FEV1 values (SMD = 0.70; 95% CI: 0.33 to 1.06; *p* = 0.0002) (Figure 5(a)). Similarly, subgroup analysis demonstrated higher FEV1 values among patients who had received pirfenidone during late‐stage COVID‐19 (SMD = 0.74; 95% CI: 0.34 to 1.15; *p* = 0.0003) (Figure 5(a)). However, the effect of pirfenidone on FEV1 during early‐stage COVID‐19 was difficult to estimate for the sole study listed (SMD = 0.51; 95% CI: −0.32 to 1.33; *p* = 0.23) (Figure 5(a)).

Figure 5Comparisons of FEV1 and ΔFEV1 between pirfenidone and glucocorticoid groups.

**Figure:**
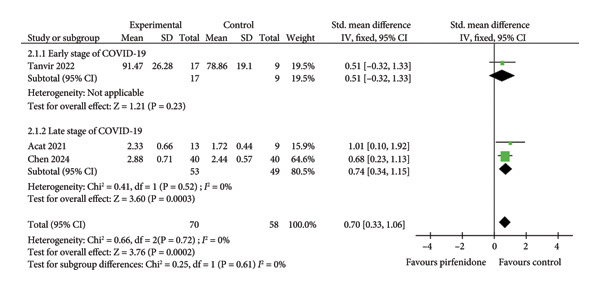
(a)

**Figure:**
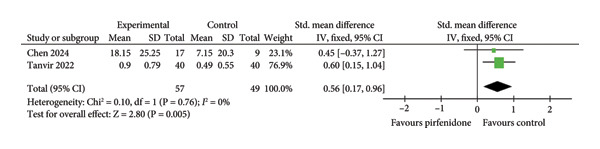
(b)

Two studies [24, 25] compared the absolute changes in FEV1 level (ΔFEV1) between experimental (57 patients) and control (49 patients) groups. A data extraction Excel calculation table was constructed to compute the absolute standard change of FEV1 from the baseline value. The value of ΔFEV1 was significantly increased with low heterogeneity (*I* ^2^ = 0%, *p* = 0.76) in the experimental group compared to the control group (SMD = 0.56; 95% CI: 0.17 to 0.96; *p* = 0.005) (Figure 5(b)).

### 3.4. Adverse Events

Three studies [21, 22, 26] enrolling 193 patients in the pirfenidone group and 163 patients in the control group reported the incidence of gastrointestinal adverse events. Because of low interstudy heterogeneity (*I* ^2^ = 0%, *p* = 0.38) (Figure 6(a)), the fixed effect model was employed to combine the data. Pirfenidone therapy was associated with a higher incidence of gastrointestinal adverse events as compared to the control group (RR = 1.82; 95% CI: 1.15 to 2.89; *p* = 0.01) (Figure 6(a)). Four studies [20, 22, 24, 26] enrolling 204 patients in the pirfenidone group and 168 patients in the control group reported the incidence of drug discontinuation due to adverse events. Because of medium interstudy heterogeneity (*I* ^2^ = 57%, *p* = 0.07) (Figure 6(b)), the random effect model was employed to combine the data. Pirfenidone therapy was associated with a higher incidence of discontinuation of therapy due to adverse events as compared to the control group (RR = 3.50; 95% CI: 1.39 to 8.81; *p* = 0.008) (Figure 6(b)). Notably, the included studies did not report serious adverse events, irreversible injuries, or fatal complications caused by pirfenidone.

Figure 6Comparisons of gastrointestinal adverse events and discontinuation between pirfenidone and control groups.

**Figure:**
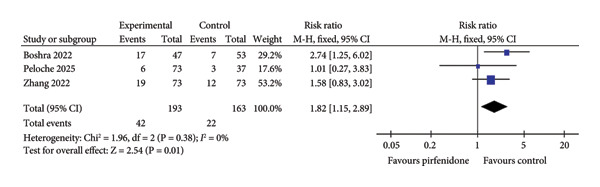
(a)

**Figure:**
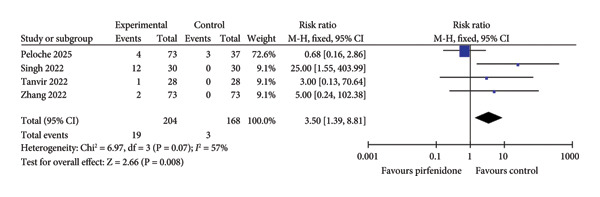
(b)

## 4. Discussion

A prospective study of 114 survivors of severe COVID‐19 revealed that over two‐thirds exhibited abnormal chest CT findings at six‐month follow‐up. Common radiographic signs included ground‐glass opacities, interstitial thickening, honeycombing, parenchymal bands, and traction bronchiectasis [5]. Further follow‐up of the same cohort demonstrated that all (100%) of the participants with fibrotic ILAs on six‐month CT scans exhibited persistent radiographic findings at 1 year, indicating chronic post‐COVID‐19 pulmonary disease [6]. Moreover, approximately half of hospitalized COVID‐19 survivors experienced postdischarge impairments of physical function and activities of daily living [27].

Viral infection and consequent hyperinflammation cause the lung injuries that complicate COVID‐19 [28]. Proinflammatory cytokine storm and overactive fibroblasts during SARS‐CoV‐2 infection may stimulate excessive collagen deposition in interstitial spaces, resulting in ILD and the consequent radiographic signs of ILA [29]. Similar pathogenic mechanisms of pulmonary fibrosis are shared between IPF and COVID‐19–associated fibrosis, suggesting a potential role of pirfenidone therapy to prevent post‐COVID‐19 ILAs [30].

Pirfenidone, a novel antifibrotic drug, exerts anti‐inflammatory, antifibrotic, and antioxidant activities and is effective in the treatment of both IPF and connective tissue disease–associated ILD [31, 32]. Pirfenidone is thought to mitigate post‐COVID‐19 lung abnormalities by inhibiting the deposition of collagen and extracellular matrix, reducing oxidative stress, and attenuating fibrosis [33]. However, because IPF is a heterogeneous disease and ILAs have resolved spontaneously in some COVID‐19 patients, the role of pirfenidone in the treatment of patients with ILAs caused by COVID‐19 remains controversial.

The present study demonstrated that pirfenidone significantly decreased chest HRCT scores in patients with post‐COVID‐19 ILAs at both early and late disease stages and significantly improved FEV1 and the absolute standard change of FVC, which are the key lung function parameters, suggesting that pirfenidone treatment may mitigate COVID‐19–associated lung injury and thereby potentially alleviate symptoms and improve quality of life. Furthermore, our study suggested a trend toward decreased all‐cause mortality among pirfenidone‐treated patients, although the trend did not reach statistical significance. It has been reported that oxygenation indices pose prognostic values in hypoxemic COVID‐19 patients; however, pirfenidone showed no significant effect on oxygenation in our study [34].

We also observed that pirfenidone significantly reduced TNF‐α levels, indicating its anti‐inflammatory role in the treatment of COVID‐19. The cytokine storm provoked by SARS‐CoV‐2 infection often causes diffuse alveolar damage and myofibroblast proliferation that are associated with collagen and extracellular matrix deposition [35]. Autopsy findings in COVID‐19 patients include bilateral interstitial pneumonia with cellular fibromyxoid exudates. Mitigation of hyperinflammation and fibrosis during the acute‐phase COVID‐19 may facilitate the resolution of pneumonitis, lower the risk of developing pulmonary fibrosis, and reduce the severity of post‐COVID‐19 chronic lung disease. Further clinical and basic research studies are needed to confirm these hypotheses.

Corticosteroid treatment may improve clinical, physiological, and radiological outcomes of COVID‐19 [14, 15]. Notably, our study found that pirfenidone therapy with or without corticosteroid coadministration reduced inflammatory cytokine levels and HRCT findings and improved pulmonary function to a greater degree than corticosteroid treatment alone. These results, derived from indirect comparisons, suggest that pirfenidone treatment provides additional benefits that may facilitate improvement in health‐related quality of life among survivors of severe COVID‐19 to some extent. A multicenter, large‐scale, and head‐to‐head trial between pirfenidone and corticosteroids in the future is necessary.

Our analysis associated pirfenidone with an increased incidence of gastrointestinal side effects due to adverse events, which is consistent with previous findings [32, 36]. A Phase 3 trial of 555 patients with IPF showed that gastrointestinal adverse events that included diarrhea, nausea, vomiting, and anorexia were more common in the pirfenidone group compared to the control group [10]. As described in the included studies, the adverse effects of pirfenidone were of mild to moderate severity and could be mitigated by dose reduction or interruption of therapy. The most common adverse event leading to discontinuation was the passage of loose stools. No serious or fatal adverse events were reported, further confirming the safety and tolerability of pirfenidone in the treatment of COVID‐19.

This is the latest and most comprehensive meta‐analysis designed to evaluate the effects of pirfenidone on lung function, imaging findings, and the risk of adverse events. However, several limitations of this meta‐analysis must be acknowledged. First, the primary limitation is the scarcity of studies regarding the effects of pirfenidone in the treatment of COVID‐19–associated ILAs, which may affect the generalizability of our findings. Second, heterogeneity was observed across some studies, which may be attributed to variations in inclusion and exclusion criteria, study duration, and time nodes for the selection of observation indicators. Although we employed random‐effects models and conducted sensitivity analyses to assess the robustness of the findings, these factors nonetheless introduce a degree of uncertainty and highlight the need for cautious interpretation. Additional high‐quality RCTs with large sample sizes and standardized outcome measures are necessary to draw robust conclusions. Third, the included studies featured the use of varied therapeutic regimens in the control groups, which introduced high heterogeneity to our analysis. Subgroup analysis based on therapeutic regimens was conducted to reduce the impact of potential confounding factors. Finally, the clinical symptom alleviation (such as cough, chest tightness, and fatigue), carbon monoxide diffusing capacity changes, and the detailed adverse event of digestive system during pirfenidone therapy have not been presented in our study due to the information loss in the enrolled studies. Despite these limitations, we have preliminarily confirmed the effectiveness, advantages over corticosteroid therapy, and safety profile of pirfenidone in the mitigation of COVID‐19–induced ILAs.

## 5. Conclusions

Our study indicated that pirfenidone was effective in preventing the onset and/or progression of ILAs caused by COVID‐19 during early‐ and late‐stage disease and exhibited a profile of manageable adverse effects as expected. Furthermore, pirfenidone therapy seemed more beneficial than glucocorticoid treatment for the mitigation of ILAs. Therefore, pirfenidone should be considered to prevent the onset or progression of post‐COVID‐19 ILAs.

## Ethics Statement

The study was approved by the Ethics Committee of the First Affiliated Hospital of Soochow University, and informed consent was waived because we did not intervene in the diagnosis or treatment of patients in this study.

## Consent

Please see the Ethics Statement.

## Disclosure

All authors approved the final version for submission.

## Conflicts of Interest

The authors declare no conflicts of interest.

## Author Contributions

Ziyi Zhang developed the graphs and wrote the manuscript. Xiang Fang, Heming Sun, and Jinhui Gao were responsible for literature search, data extraction, and statistical analysis. Ting Xu and Jiajia Wang conceived and designed the study. Finally, Jiajia Wang supervised the study. Ziyi Zhang, Xiang Fang, Jinhui Gao, and Heming Sun contributed equally to this work. Jiajia Wang is the first corresponding author for this study.

## Funding

Funding for this work was supported by the Gusu Health Talent of Suzhou City (No. GSWS202206), Hui‐Chun Chin and Tsung‐Dao Lee Chinese Undergraduate Research Endowment (CURE), “Ruan Changgeng” Research and Innovation Fund Project for Graduate Student, Boxi Clinical Research Project (No. BXLC2024014), and Jiangsu Provincial Medical Key Discipline (No. ZDXK202201).

## Supporting Information

Additional supporting information can be found online in the Supporting Information section.

Supporting Table S2: Quality assessment of cohort studies.

Supporting Figure S2: Assessments of each risk of bias item shown as percentages across all included studies.

Supporting Figure S3: Risk of bias for four randomized controlled trials (RCTs).

Supporting Figure S4: Comparisons of SpO_2_ and ΔSpO_2_ between the pirfenidone and control groups.

Supporting Figure S5: Comparisons of length of hospital stay between the pirfenidone and control groups.

Supporting Figure S6: Comparisons of all‐cause mortality between the pirfenidone and control groups.

## Supporting information


**Supporting Information 1** Supporting Table S1: Search strategies.

**Supporting Information 2** Supporting Figure S1: Flowchart of literature selection process.

## Data Availability

Data from the studies are already available in publications.
