# Supplementary material for: Efficacy and Safety of Pirfenidone for Mitigation of Interstitial Lung Abnormalities in COVID‐19 Patients: A Meta‐Analysis
Source: Can Respir J. 2026 Jan 10;2026:8812779. doi: 10.1155/carj/8812779 (PMC12789973; doi:10.1155/carj/8812779)
Supplement: Supplementary file 1 — Supporting Information 1 Supporting Table S1: Search strategies. [file CARJ-2026-8812779-s002.docx]

# Supplemental materials

**Table S1**: Search strategies for all databases

| PubMed (97 items) | |
| --- | --- |
| #1 | COVID-19[MeSH Terms] |
| #2 | (COVID-19[Title/Abstract]) OR (2019-nCoV Infection[Title/Abstract]) OR (SARS-CoV-2 Infection[Title/Abstract]) OR (2019 Novel Coronavirus Disease[Title/Abstract]) OR (COVID-19 Virus Infection[Title/Abstract]) OR (Coronavirus Disease 2019[Title/Abstract]) OR (Severe Acute Respiratory Syndrome Coronavirus 2 Infection[Title/Abstract]) OR (2019-nCoV Disease[Title/Abstract]) OR (COVID-19 Pandemic[Title/Abstract]) |
| #3 | #1 OR #2 |
| #4 | Pulmonary Fibrosis[MeSH Terms] |
| #5 | (pulmonary fibrosis[Title/Abstract]) OR (lung fibrosis[Title/Abstract]) OR (interstitial lung disease[Title/Abstract]) OR (ILD[Title/Abstract]) OR (ILDs[Title/Abstract]) OR (interstitial lung diseases[Title/Abstract]) |
| #6 | #4 OR #5 |
| #7 | Antifibrotic Agents[MeSH Terms] |
| #8 | (antifibrotic agents[Title/Abstract]) OR (Pirfenidone[Title/Abstract]) OR (deskar[Title/Abstract]) OR (esbriet[Title/Abstract]) |
| #9 | #8 OR #9 OR #10 |
| #10 | #3 AND #6 AND #9 |
| Embase (406 items) | |
| #1 | 'coronavirus disease 2019'/exp |
| #2 | 'covid-19':ti,ab,kw OR '2019-ncov infection':ti,ab,kw' OR 'sars-cov-2 infection':ti,ab,kw OR '2019 novel coronavirus disease':ti,ab,kw OR 'covid-19 virus infection':ti,ab,kw OR 'coronavirus disease 2019':ti,ab,kw OR 'severe acute respiratory syndrome coronavirus 2 infection':ti,ab,kw OR '2019-ncov disease':ti,ab,kw OR 'covid-19 pandemic':ti,ab,kw |
| #3 | #1 OR #2 |
| #4 | 'lung fibrosis'/exp |
| #5 | 'interstitial lung disease':ti,ab,kw OR 'interstitial lung diseases':ti,ab,kw' OR 'ild':ti,ab,kw OR 'ilds':ti,ab,kw OR 'pulmonary fibrosis':ti,ab,kw OR 'lung fibrosis':ti,ab,kw' |
| #6 | #4 OR #5 |
| #7 | 'antifibrotic aent'/exp |
| #8 | 'antifibrotic agents':ti,ab,kw OR 'pirfenidone':ti,ab,kw OR 'deska':ti,ab,kw OR 'esbriet':ti,ab,kw |
| #9 | #7 OR #8 |
| #10 | #3 AND #6 AND #9 |
| Cochrane Library (42 items) | |
| #1 | MeSH descriptor: [COVID-19] explode all trees |
| #2 | 'covid-19':ti,ab,kw OR '2019-ncov infection':ti,ab,kw' OR 'sars-cov-2 infection':ti,ab,kw OR '2019 novel coronavirus disease':ti,ab,kw OR 'covid-19 virus infection':ti,ab,kw OR 'coronavirus disease 2019':ti,ab,kw OR 'severe acute respiratory syndrome coronavirus 2 infection':ti,ab,kw OR '2019-ncov disease':ti,ab,kw OR 'covid-19 pandemic':ti,ab,kw |
| #3 | MeSH descriptor: [Pulmonary Fibrosis] explode all trees |
| #4 | 'interstitial lung disease':ti,ab,kw OR 'interstitial lung diseases':ti,ab,kw' OR 'ild':ti,ab,kw OR 'ilds':ti,ab,kw OR 'pulmonary fibrosis':ti,ab,kw OR 'lung fibrosis':ti,ab,kw' |
| #5 | MeSH descriptor: [antifibrotic agents] explode all trees |
| #6 | 'antifibrotic agents':ti,ab,kw OR 'pirfenidone':ti,ab,kw OR 'deska':ti,ab,kw OR 'esbriet':ti,ab,kw |
| #7 | #1 OR #2 |
| #8 | #3 OR #4 |
| #9 | #5 OR #6 |
| #10 | #7 AND #8 AND #9 |
| Web of Science (105 items) | |
| #1 | TS=(COVID-19 OR 2019-nCoV Infection OR SARS-CoV-2 Infection OR 2019 Novel Coronavirus Disease OR COVID-19 Virus Infection OR Coronavirus Disease 2019 OR Severe Acute Respiratory Syndrome Coronavirus 2 Infection OR 2019-nCoV Diseas OR COVID-19 Pandemic) |
| #2 | TS=(interstitial lung disease OR interstitial lung diseases OR ILD OR ILDs OR pulmonary fibrosis OR lung fibrosis) |
| #3 | TS=(Antifibrotic agents OR Pirfenidone OR deska OR esbriet ) |
| #4 | #1 AND #2 AND #3 |
| CBM (12 items) | |
| #1 | "新型冠状病毒肺炎"[不加权:扩展] |
| #2 | ("2019新型冠状病毒感染疾病"[常用字段:智能] OR "新型冠状病毒感染肺炎;"[常用字段:智能] OR "2019新型冠状病毒感染肺炎"[常用字段:智能] OR "2019新型冠状病毒感染"[常用字段:智能] OR "2019新冠肺炎"[常用字段:智能]) |
| #3 | #1 OR #2 |
| #4 | "肺纤维化"[不加权:扩展] |
| #5 | ("肺泡炎"[常用字段:智能] OR "纤维化肺泡炎"[常用字段:智能] OR "肺纤维化"[常用字段:智能]) |
| #6 | #4 OR #5 |
| #7 | "药用制剂"[不加权:扩展] |
| #8 | ("抗纤维化药物"[常用字段:智能] OR "吡非呢酮"[常用字段:智能]) |
| #9 | #7 OR #8 |
| #10 | #3 AND #6 AND #9 |
| CNKI (63 items) | |
| #1 | 新冠肺炎 + '新冠肺炎(covid-19)' + 新冠肺炎患者 + 新冠肺炎疫情 + 新冠肺炎疫情影响 |
| #2 | 肺纤维化 + '肺纤维化(pf)' + 肺纤维化治疗 + 肺纤维化疾病 + 肺纤维化发病机制 |
| #3 | 抗纤维化 + 抗纤维化作用 + 抗纤维化治疗 + 抗纤维化疗效 + 抗纤维化药物 + 抗纤维化治疗策略 |
| #4 | #1 AND #2 AND #3 |
| Weipu (325 items) | |
| #1 | 新冠肺炎 + '新冠肺炎(covid-19)' + 新冠肺炎患者 + 新冠肺炎疫情 + 新冠肺炎疫情影响 |
| #2 | 肺纤维化 + '肺纤维化(pf)' + 肺纤维化治疗 + 肺纤维化疾病 + 肺纤维化发病机制 |
| #3 | 抗纤维化 + 抗纤维化作用 + 抗纤维化治疗 + 抗纤维化疗效 + 抗纤维化药物 + 抗纤维化治疗策略 |
| #4 | #1 AND #2 AND #3 |
| WanFang (72 items) | |
| #1 | 新冠肺炎 OR 新冠肺炎患者 OR 新冠肺炎疫情 OR 新冠肺炎疫情影响 |
| #2 | 肺纤维化 OR 肺纤维化治疗 OR 肺纤维化疾病 OR 肺纤维化发病机制 |
| #3 | 抗纤维化 OR 抗纤维化作用 OR 抗纤维化治疗 OR 抗纤维化疗效 OR 抗纤维化药物 OR 抗纤维化治疗策略 |
| #4 | #1 AND #2 AND #3 |

CBM: China Biology Medicine; CNKI: China National Knowledge Infrastructure

| **Cohort Studies** | | | | | | | | | |
| --- | --- | --- | --- | --- | --- | --- | --- | --- | --- |
| Study | Representativeness of the exposed cohort | Selection of the non-exposed cohort | Ascertainment of exposure | Demonstration that outcome of interest was not present at start of study | Comparability of cohorts on the basis of the design or analysis | Assessment of outcome | Was follow-up long enough for outcomes to occur | Adequacy of follow up of cohorts | Quality Score |
| Acat et al | 0 | ★ | ★ | ★ | ★ | ★ | 0 | ★ | 6 |
| Singh et al | 0 | ★ | ★ | ★ | ★ | ★ | ★ | ★ | 7 |
| Banerjee et al | 0 | ★ | ★ | ★ | ★ | ★ | ★ | ★ | 7 |

**Table S2:** Assessment of included cohort studies by Newcastle-Ottawa Scale (NOS)

Note: A study can be awarded a maximum of one star for each numbered item within the selection and exposure categories. A maximum

of two stars can be given for comparability.
