# Supplementary material for: Efficacy and Safety of Pirfenidone for Mitigation of Interstitial Lung Abnormalities in COVID‐19 Patients: A Meta‐Analysis
Source: Can Respir J. 2026 Jan 10;2026:8812779. doi: 10.1155/carj/8812779 (PMC12789973; doi:10.1155/carj/8812779)
Supplement: Supplementary file 2 — Supporting Information 2 Supporting Figure S1: Flowchart of literature selection process. [file CARJ-2026-8812779-s001.docx]

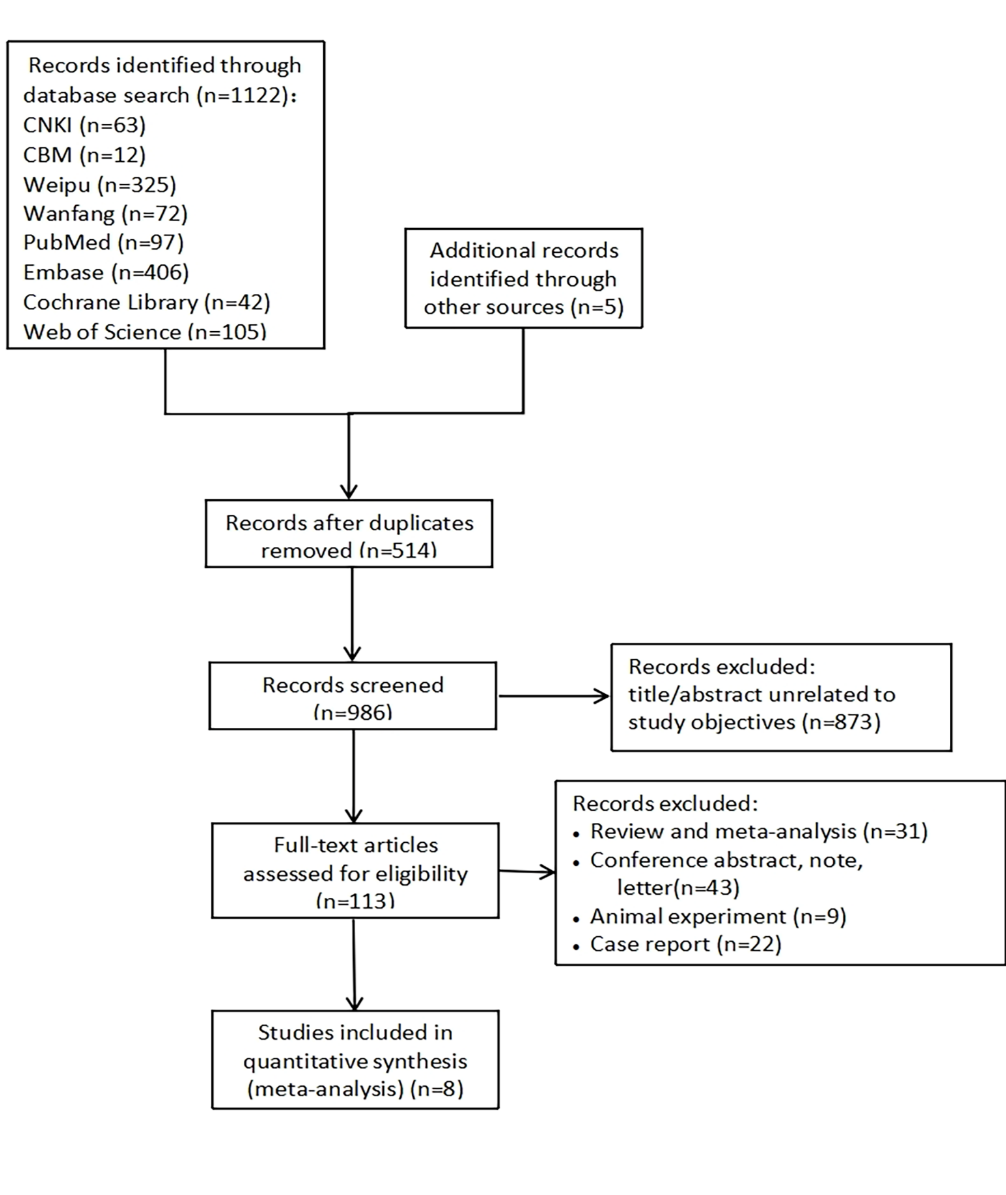


**Figure S1.** Flow chart of literature selection process.


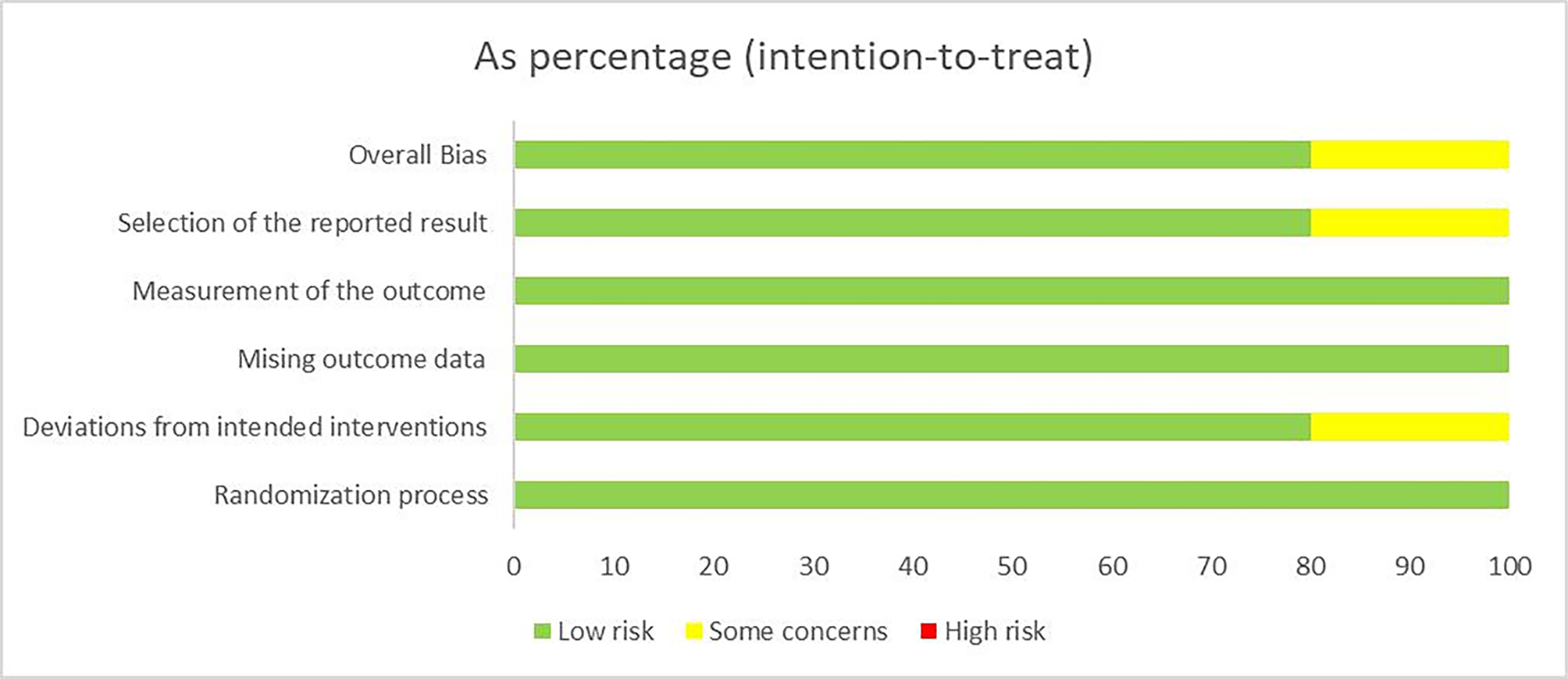


**Figure S2.** Risk of bias summary. Assessments of each risk of bias item shown as percentages across all included studies.


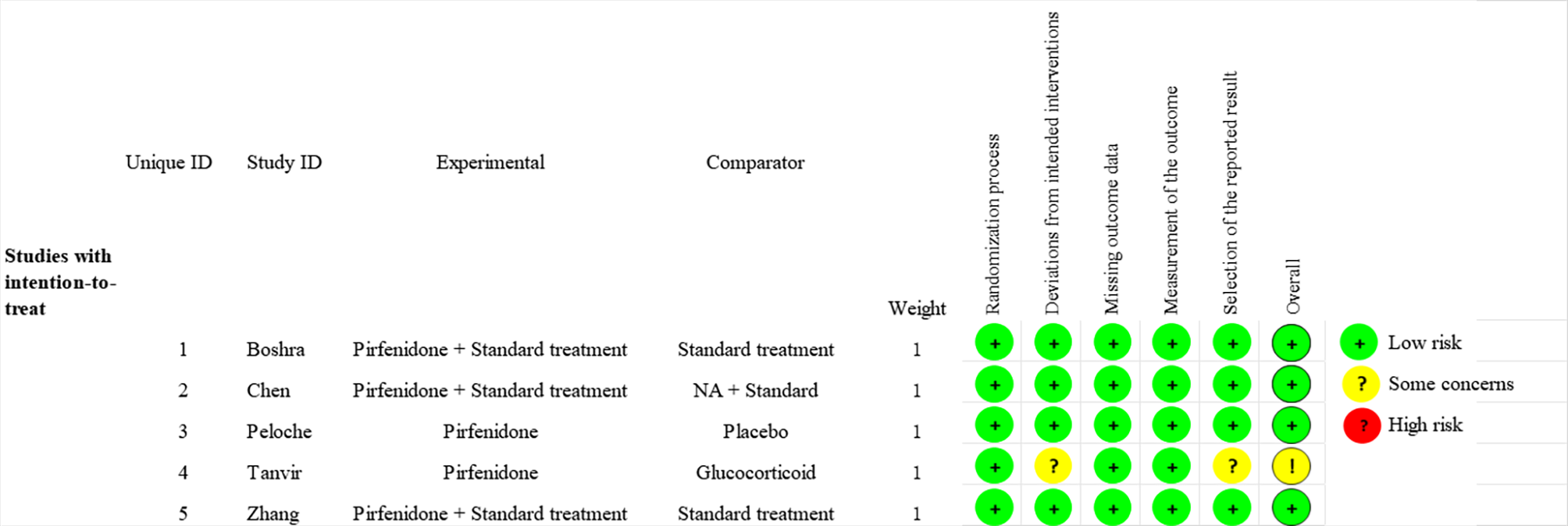


**Figure S3.** Risk of bias for four randomized controlled trials (RCTs).


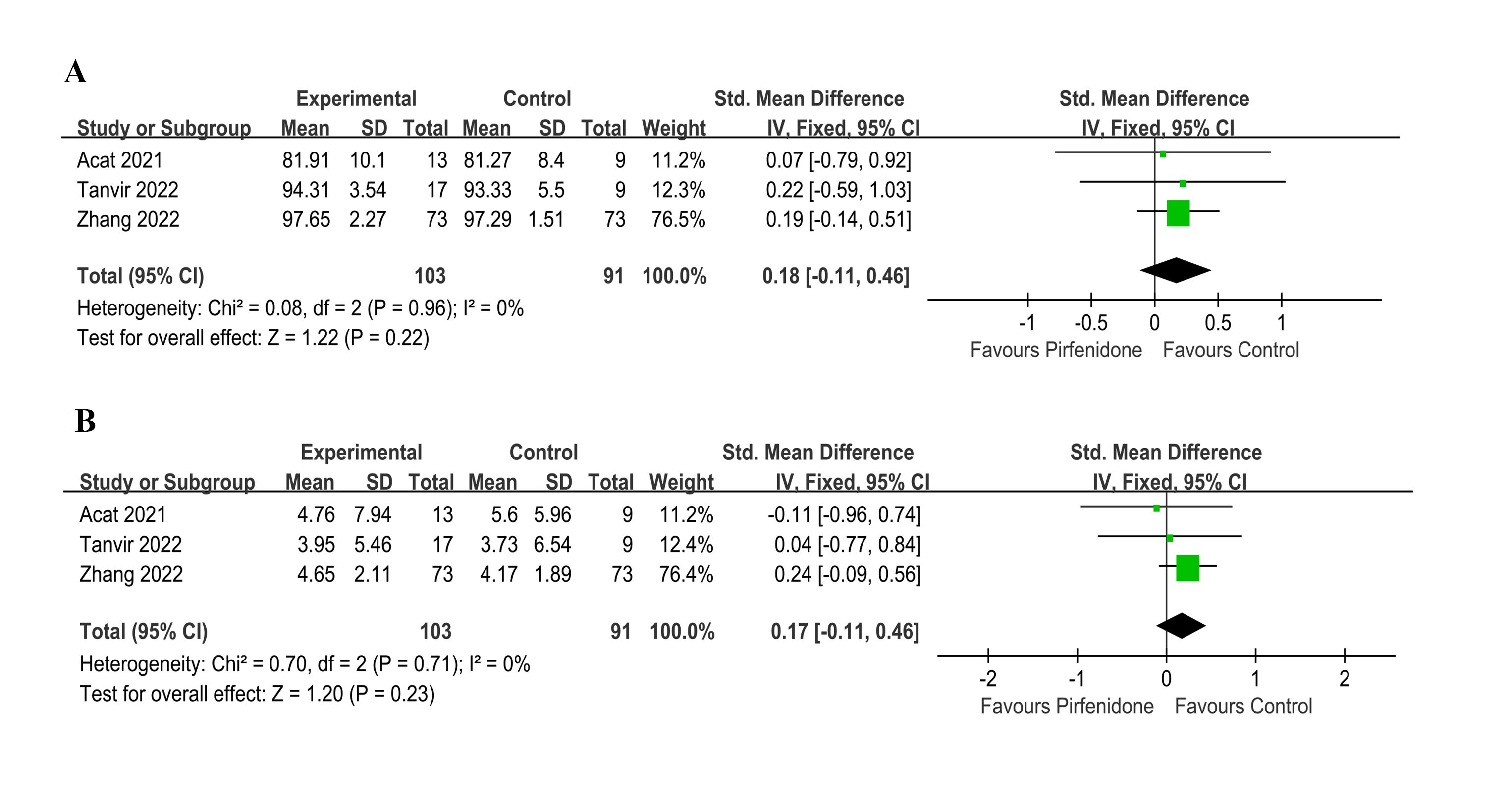


**Figure S4.** Comparisons of SpO2 and ΔSpO2 between pirfenidone and control groups.


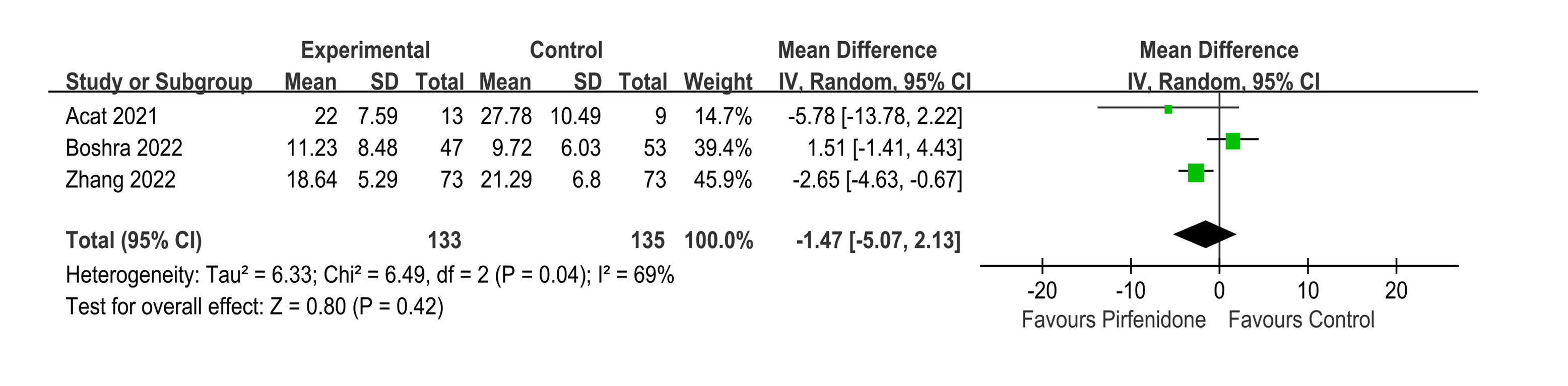


**Figure S5.** Comparison of length of hospital stay between pirfenidone and control groups.


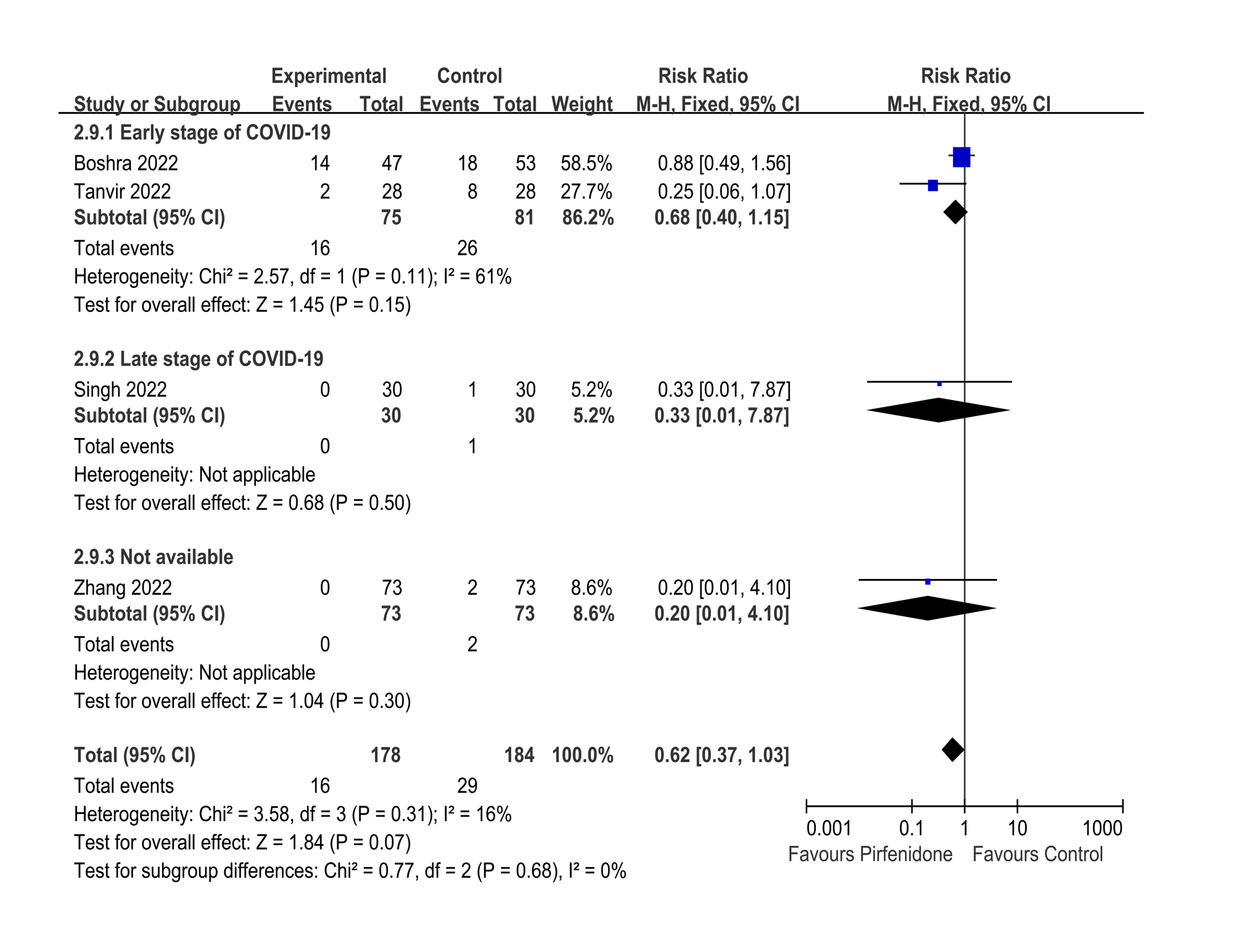


**Figure S6.** Comparison of all-cause mortality between pirfenidone and control groups.
